# Supplementary material for: Responses of candidate intensity measures to different mental and motor load levels using upper limb exergames in typically developing children and adolescents
Source: PLoS One. 2025 Jun 25;20(6):e0326371. doi: 10.1371/journal.pone.0326371 (PMC12193600; doi:10.1371/journal.pone.0326371)
Supplement: Attachment — Submitted filename: Answer Reviewers.docx [file pone.0326371.s002.docx]

Answer to reviewer 1:

We want to thank the reviewer for the critical yet constructive and useful feedback. We have responded to each comment and tried to clarify several points that might have been unclear in the first version. We hope the reviewer agrees with these changes.

Reviewer #1: The purpose of this is study is to investigate the response of physiological variables such as heart rate variability (HRV), skin conductance (SC), activity counts and movement repetitions normalized for the maximal capacity (%ACmax and %MOVmax, respectively), and cognitive variables as the NASA-TLX scale to different mental and motor intensity levels of two self-developed upper limb exergames in typically developing children. The authors also investigated the effects of age on the responses of the measures.

The article is interesting, but the relevance and applicability is not very clear. Here are my suggestions:

- The purpose should be clearly stated, avoiding excessive use of acronyms.
- The purpose in the abstract differs from the one presented in the main text.

Thank you for this comment. To clarify the relevance of this work, we changed the background in the abstract and added a paragraph clarifying the overall importance of our work. Furthermore, we formulated the objectives in the abstract and introduction now almost similarly. Unfortunately, we left the abbreviations in the abstract in the purpose to remain within the maximal word count as prescribed by the journal. Through the text, we avoided using acronyms at the beginning of the sentences, and left them only in the cases were the concepts were repeated more than three times (following guidelines). We hope the reviewers agrees on this decision.

As we state in the abstract (lines 45-49) with “Objective: To investigate the response of heart rate variability (HRV), skin conductance (SC), activity counts and movement repetitions normalized for the maximal capacity (%ACmax and %MOVmax, respectively), and the NASA-TLX scale to different mental and motor intensity levels of two self-developed upper limb exergames in typically developing children” and in the introduction (lines 134-140) “we investigated how HRV, SC, %ACmax, %MOVmax, and the NASA-TLX responded to personalized motor and mental intensity levels of upper limb exergames” we investigated the responses of the several measures to changes in exercise intensity.

Although this is the objective of our investigation, we agree with the reviewer that this is not our end goal but rather the means to understand how to capture better therapy intensity when considering its multidimensional nature. We have adapted slightly the abstract to match the introduction and for clarity.

Introduction:
- The introduction provides detailed information on different types of therapy and their outcomes. However, there is no mention or explanation regarding exergames in the introduction, or how the results from this study can help the therapy procedures.

First, we agree with the reviewer that we focused on the measures and did not explain to the reader why better measures are needed and how this would help the field of neurorehabilitation. Therefore, we have included several important reasons in the introduction. However, this is a much more complex topic, which we discuss in greater detail in Goikoetxea & van Hedel (Defining, quantifying, and reporting intensity, dose, and dosage of neurorehabilitative interventions focusing on motor outcomes. Front. Rehabil. Sci., 2023, doi: 10.3389/fresc.2023.1139251).

Second, we used exergames because this would allow us to systematically investigate the responses of the various measures to changes in motor or mental load (we added this also in the methods section under Par. 2.3), and we did not intend to analyze their effectiveness. In a previous paper, we tried to investigate the effect of different levels of upper limb robotic therapy in a very pragmatic approach (Goikoetxea-Sotelo G, van Hedel HJA. Responses of several measures to different intensity levels of upper limb exergames in children with neurological diagnoses: a pilot study. Front Rehabil Sci. 2024;5:1405304. doi: 10.3389/fresc.2024.1405304.) but we noted that therapists used different approaches to increase task intensity, i.e., increasing motor and/or mental load). Therefore, in this study, we created two exergames that would allow us to better distinguish between increasing mental and motor load.

After this study it is too early for us to clarify how well the results can help therapy procedures. We are currently using the same protocol in young patients and testing the reliability of the measures. In addition, we have started by applying the measures during regular occupational and upper limb robotic therapy sessions. At the end of these studies, we would need less speculation on how the measures could prove valuable for therapy procedures.

- The significance of exergames and their relevance to the study's purpose should be addressed. It is unclear how exergames influence the therapy in question.

The use of the exergames is the means to understand how to measure therapy intensity better rather than to assess the effectiveness of these. We shortly explained the added value of using exergames in the methods section (Par. 2.3). In addition, we slightly changed the title to improve the clarity of this point.

Methods:

Figure 1 please does not identify the patient.

We have anonymized the patient as requested.

On page 9, reference number should be added for line 205.

The reference is placed at the end of the sentence, line 207 reference number 33, we have now placed it right after “Aponte and colleagues”.

The sample rate for the Polar system needs to be described.

We have added this information in the methods section under “Heart rate variability”.

Additionally, the heart rate units used are uncommon and make comparisons with other studies challenging.

We do not provide any heart rate information. The unit we provide refers only to heart rate variability and is RMSSD/RR (Root mean squared of successive differences/r-to-r ratio), as suggested by Sacha & Pluta (2005, J. Electrocardiology doi: 10.1016/j.jelectrocard.2004.09.015, and 2008, International Journal of Cardiology, doi: 10.1016/j.ijcard.2007.06.047). This can also be found in Tian et al. (2017, Front Psychol, doi: 10.3389/fpsyg.2017.01121) or our recently published paper Goikoetxea-Sotelo and van Hedel (2024, Front Rehab Sci, doi: 10.3389/fresc.2024.1405304), which are the only studies until the date we could find that used heart rate variability for this purpose.

Publications analyzing HRV at rest indeed used RMSSD data (without normalization). Although we also computed RMSSD data, we were aware that most of the variability in the RMSSD data came from the mathematical relationship between RMSSD and HR (see Sacha & Pluta, 2005, J. Electrocardiology and 2008, International Journal of Cardiology). By normalizing the RMSSD with the mean RR, we subtract the effects of HR from the equation; thus, the differences we observe should represent solely the CNS activation (theoretically).

Provide more details about the NASA TLX questionnaire, including at least one more paragraph.

We added the NASA-TLX document as a supplementary material (both the English version and the German version). We additionally emphasized on it psychometric properties by referring to relevant literature in the methods section 2.4.4. We avoided adding a whole paragraph to avoid repetition, as we already have a whole paragraph in the introduction discussing the NASA-TLX and its properties.

A more precise explanation of its development and contents can be found under Hart & Staveland (1988, Hancock, P. A. & Meshkati, N., doi: 10.1016/S0166-4115(08)62386-9) or specifically for children under Laurie-Rose et al. (2014, Am J Psychol., doi: 10.5406/amerjpsyc.127.1.0107)

Results:
Variables in Table 1 should include their corresponding units.

Thank you very much for noticing this, we have added the units accordingly.

Figures 6 and 7 are difficult to interpret due to poor resolution.
Thank you for this comment. We believe the problem lies on the review platform, as the original pictures we uploaded are of very high resolution (we added them at the end of this document, so that the reviewer can better interpret them). If this still remains a problem, we will try to solve this problem with the editors.

General comments:
The sample size is small, especially when divided into three age groups. Based on motor development, the sample size is insufficient to support strong conclusions.

We agree that the number per age group is rather small. Therefore, we did not investigate the effect of age by comparing the three age groups, we included the data of all participants in a regression model (we used age as a continuous variable for our regression model (in contrast to the intensity levels with repeated measures), meaning that the sample size for the analysis remains 21).

We mentioned the three age groups across lines 143-146 refers to clarify our efforts to include participants in all stages of development and, therefore, have a good representation of the whole childhood and adolescence. We wanted to avoid having only participants from, for example, the final stages of cognitive development, i.e., 13-17 years, as these participants might respond differently to the measures (especially to the NASA-TLX) compared to younger ones.

In addition, as we mention in the limitation section (lines 609-612) and the conclusion (lines 645-648), this is a first study with typically developing children and the results should be confirmed in the target population, i.e., children with neurological diagnoses. There, a larger sample size (at least 30 participants if we follow psychometric property guidelines) would be needed.

Lastly, the discussion does not clearly explain the relationship between exergames and the therapy.

As we commented before, we selected the use of exergames to systematically investigate our research question as it would allow us to control several parameters that would not be possible during regular conventional therapeutic approaches. We did not intend to extrapolate from our findings directly to the clinical application.

Therefore, we included in our limitations: “In addition, the present study focused on two specific exergames, and the participants were typically developing children and adolescents. The results are specific to these games, participants, and study settings. Future studies with larger cohorts of the actual target group, i.e., children and adolescents with neurological diagnoses undergoing upper limb neurorehabilitation, e.g., occupational therapy, could confirm our findings in an ecologically valid way. Future studies should also assess the psychometric properties, e.g., the test-retest reliability of these candidate intensity measures in the target group.”

The study can be very interesting, but does not show clearly its applicability for therapy purposes. For example, what is the importance of HRV increase or decrease in therapy? How 3min gaming practice can improve children skills in the long term?

Thank you very much for this feedback. As we state in our objectives, the goal of this investigation is to have a first impression on how the measures react to changes in motor and mental intensity. Therefore, it might be too early to withdraw clear conclusion on their applicability for therapy. However, we agree that, as the applicability for therapy would be a future goal, it makes sense to hypothesize on their potential applicability. We have included some information about the applicability of the measures in a separate section. Especially for HRV, we have performed additional analyses to try to understand how it could be used. However, the results are somewhat difficult to interpret, and no clear conclusions could be withdrawn.
