# Supplementary material for: Responses of candidate intensity measures to different mental and motor load levels using upper limb exergames in typically developing children and adolescents
Source: PLoS One. 2025 Jun 25;20(6):e0326371. doi: 10.1371/journal.pone.0326371 (PMC12193600; doi:10.1371/journal.pone.0326371)
Supplement: Attachment — Submitted filename: Response_Reviewers.docx [file pone.0326371.s003.docx]

Reviewer #1: I would like to thank the authors for the very detailed answers on the response to my comments. It is much more clear now the purpose of the work. The figure added in the review is very clear as well.

We would like to thank the reviewer for the previous and this last remarks, which we consider have improved the quality of our manuscript.

some minor comments:
- Line 69, the sentence does not look complete.

We have adapted this sentence for clarity.

- Although mentioned the NASA-TLX questionnaire as a supplementary material, in the pdf I have access, it does not show. I suggest the authors to upload it in a repository, same as you did with the games version.

We suppose this is an error from the system, as we can see the document in the uploads. We have, however, added the NASA-TLX document to the repository as suggested.
